# EARLY POSTOPERATIVE OUTCOMES OF THE ESOPHAGECTOMY MINIMALLY INVASIVE IN ESOPHAGEAL CANCER

**DOI:** 10.1590/0102-672020230025e1743

**Published:** 2023-07-10

**Authors:** Thiago Francischetto, Vaner Paulo da Silva Fonseca Pinheiro, Eduardo Freitas Viana, Eduardo Dias de Moraes, Bruno Mendonça Protásio, Marco Antônio Oliveira Lessa, Gustavo Lousado de Almeida, Victor Rivera Duran Barretto, Alexandre Farias de Albuquerque

**Affiliations:** 1 Aristides Maltez Hospital, Bahia League Against Cancer – Salvador (BA), Brazil; 2 Universidade Federal da Bahia, Bahia School of Medicine – Salvador (BA), Brazil; 3 Santa Casa de Misericórdia da Bahia, Santa Izabel Hospital – Salvador (BA), Brazil.

**Keywords:** Esophageal neoplasms, Esophagectomy, Minimally invasive surgical procedures, Morbidity, Mortality, Neoplasias esofágicas, Esofagectomia, Procedimentos cirúrgicos minimamente invasivos, Morbidade, Mortalidade

## Abstract

**BACKGROUND:**

The incidence of esophageal cancer is high in some regions and the surgical treatment requires reference centers, with high volume, to make surgery feasible.

**AIMS:**

To evaluate patients undergoing minimally invasive esophagectomy by thoracoscopy in prone position for the treatment of esophageal cancer and to recognize the experience acquired over time in our service after the introduction of this technique.

**METHODS:**

From January 2012 to August 2021, all patients who underwent the minimally invasive esophagectomy for esophageal cancer were retrospectively analyzed. In order to assess the factors associated with the predefined outcomes as fistula, pneumonia, and intrahospital death, we performed univariate and multivariate logistic regression analyses, accounting for age as an important factor.

**RESULTS:**

Sixty-six patients were studied, with mean age of 59.5 years. The main histological type was squamous cell carcinoma (81.8%). The incidence of postoperative pneumonia and fistula was 38% and 33.3%, respectively. Eight patients died during this period. The patient's age, T and N stages, the year the procedure was performed, and postoperative pneumonia development were factors that influenced postoperative death. There was a 24% reduction in the chance of mortality each year, associated with the learning curve of our service.

**CONCLUSIONS:**

The present study presented the importance of the team's experience and the concentration of the treatment of patients with esophageal cancer in reference centers, allowing to significantly improve the postoperative outcomes.

## RESUMO

**RACIONAL:**

A incidência do câncer de esôfago é elevada em algumas regiões e o tratamento cirúrgico requer centros de referência com alto volume para viabilizar a cirurgia.

**OBJETIVOS:**

Avaliar os pacientes submetidos à esofagectomia minimamente invasiva, por toracoscopia na posição prona, para o tratamento do câncer de esôfago e conhecer a experiência adquirida ao longo do tempo em nosso serviço após a introdução desta técnica.

**MÉTODOS:**

De janeiro de 2012 a agosto de 2021, foram analisados retrospectivamente todos os pacientes submetidos à esofagectomia minimamente invasiva para câncer de esôfago. Para avaliar os fatores associados aos desfechos predefinidos de fístula, pneumonia e óbito intra-hospitalar, realizamos análises de regressão logística univariada e multivariada considerando a idade como fator importante.

**RESULTADOS:**

Foram estudados 66 pacientes, com idade média de 59,5 anos. O tipo histológico mais frequente foi carcinoma espinocelular (81,8%). A incidência de pneumonia pós-operatória e fístula foi de 38% e 33,3%, respectivamente. Oito pacientes morreram durante este período. A idade do paciente, os estágios T e N, o ano da realização do procedimento e o desenvolvimento de pneumonia pós-operatória foram fatores que influenciaram o óbito. Houve uma redução de 24% na chance de mortalidade, ano a ano, associada à curva de aprendizado do nosso serviço.

**CONCLUSÕES:**

O presente estudo mostrou a importância da experiência da equipe e da concentração do tratamento de pacientes com câncer de esôfago em centros de referência, possibilitando melhorar significativamente o resultado pós-operatório.

## INTRODUCTION

Esophageal cancer is currently the 8th most common neoplasm in the world, with an estimated 570,000 new cases in 2020. It is also the 6th neoplasm with the highest number of deaths, with approximately 510,000 deaths recorded in 2020^19^. The disease has a great geographical variation and high incidence in the East, where there is a predominance of the squamous cell carcinoma (SC), with intrathoracic location and strong association with alcohol and tobacco use. In contrast, in the United States and Europe, there is a lower incidence of this type of cancer, with a predominance of the distal esophageal adenocarcinoma (EA) located in the esophagogastric transition and more associated with risk factors such as obesity and gastroesophageal reflux disease (GERD)^7,9^.

Surgical resection is the main therapeutic modality to treat this neoplasm. When associated with neoadjuvant radiotherapy (RXT) and/or chemotherapy (CT), the surgery has the potential to offer the best survival results to patients with SC and EA with a good quality of life^1,21^. However, esophagectomy is a highly complex procedure and have a high morbidity and mortality rate. For that reason, they must be performed in high-volume reference centers with experienced multidisciplinary teams to make surgery feasible with acceptable mortality rates below 5%^2,8,11–13^.

Currently, minimally invasive techniques have been disseminated in the treatment of esophageal cancer, with studies demonstrating the safety in oncological results, a reduction in the morbidity rate, and survival rates similar to those of traditional techniques^3,12,14,16,18^. Among the main approaches, thoracoscopy in the prone position presents better ergonomy to the surgeon in the dissection of mediastinal structures, lower rate of respiratory complications, and reduction in surgical time^4–6,17^.

The minimally invasive esophagectomy (MIE) was introduced in our service in 2012 by means of the hybrid approach of thoracoscopy with the patient in the prone position.

This study aimed to retrospectively evaluate all patients submitted to MIE, by thoracoscopy in esophageal cancer and to recognize the experience acquired over time after the introduction of the technique, with special attention to the morbidity and mortality outcomes related to the surgical procedure as well as short and long-term oncological results.

## METHODS

### Patient selection

From January 2012 to August 2021, we analyzed all patients who underwent the MIE technique described above for esophageal cancer. All cases were followed up by the same surgeons at the reference center for cancer, and the data were collected retrospectively.

Preoperative diagnosis and staging were performed through esophagogastroduodenoscopy (EGD) with biopsy and computed tomography of the thorax and abdomen with contrast. Cases of esophagectomy performed by palliation or nonmalignant indications were excluded, as well as cases with metastatic disease or T4b.

### Surgical approach

Patients were submitted to esophagectomy under general anesthesia with non-selective intubation combined with peridural anesthesia. The approach started with thoracoscopy in the prone position, with slight elevation of the right hemothorax and the use of three or four portals inserted along the posterior axillary line in the 5th, 7th, and 9th right intercostal spaces. During the thoracic stage, the entire thoracic esophagus was dissected by thoracoscopy in monoblock with 107, 108, 109, 110, 111 and 112 mediastinal lymph nodes. After the thoracic stage was completed, the patient was placed in dorsal decubitus with exposure of the left cervical region.

Subsequently, the abdominal stage was initially performed by conventional approach with median supraumbilical incision, dissection of the abdominal esophagus, and a monoblock proximal gastric segment with lymph nodes levels 1, 2, 3a, 7, 8a, 9, 11p, and 19. After this stage, left cervicotomy and cervical esophagus dissection, sectioning of the esophagus, and removal of the surgical specimen by abdominal route were performed.

Reconstruction was performed with a gastric tube via the posterior mediastinum and preparation of cervical anastomosis with the remaining cervical esophageal stump. Drains were left in the right chest and left cervical. Jejunostomy or nasoenteral tube were performed for postoperative nutrition. Since 2021, the abdominal stage was also performed laparoscopically.

### Outcomes and statistical analysis

The primary outcomes of this study were fistula development, pneumonia, and death related to the surgery. The fistula was defined as a non-physiological communication between two or more structures initiated in the first seven days after surgery.

The information collected were sex, age, comorbidities (systemic arterial hypertension – SAH, diabetes mellitus – DM, smoking, alcoholism, pneumopathy), American Society of Anesthesiologists (ASA) score, lesion location, type and degree of histological differentiation, clinical tumor, node, and metastasis (TNM) staging, neoadjuvant treatment (NT), response to NT, intraoperative TNM staging, histopathological diagnosis, resected lymph nodes, length of hospital stay, morbidity, and mortality.

For descriptive purposes of baseline data, we analyzed the absolute and relative frequencies for categorical data or median with quartiles for continuous variables.

To assess factors associated with the predefined outcomes of fistula, pneumonia, and intrahospital death, we performed a univariate logistic regression analysis and a multivariate logistic regression accounting for age as a confounder. Alpha was defined as 0.05, and all analyses were performed using the software R (R Core Team, 2022).

The study was approved by the Committee on Human Research Publications and Ethics of Santa Izabel Hospital (number 5.180.063) and informed consent was obtained from all individual participants included.

## RESULTS

### Demographic and preoperative characteristics

During the period stated in the methods section, data from 66 patients were analyzed, of which 48 (72.7%) were men and 18 (27.3%) were women. The mean age of patients was 59.5 years (standard deviation±8 years). Among comorbidities, smoking accounted for 56% and alcoholism, 54.5%, followed by arterial hypertension (34.8%). The ASA score surgical risk classification was mostly II (87.8%). Demographics and comorbidities are demonstrated in Table 1.

**Table 1:** Demographic and preoperative characteristics.

			Sample (n=66) n (%)
Gender			48 (72.7)
	Male		18 (27.3)
	Female		59.3 (±8.03)
Mean age, years (SD)			23 (34.8)
Comorbidities			12 (18.1)
	SAH		3 (4.5)
	Diabetes		1 (1.5)
	Cardiopathy		37 (56)
	Pneumopathy		36 (54.5)
	Smoking		5 (7.57)
	Alcoholism		58 (87.8)
ASA Score			1 (1.5)
	I II III		2 (3)
EGD			
	Local of lesion		
		20 cm DA	2 (3)
		20 – 30 cm DA	33 (50)
		30 – 40 cm DA	17 (25.7)
		Distal esophagus	6 (9)
		EGT Siewert I	4 (6)
		EGT Siewert II	1 (1.5)
Histological type			
	Adenocarcinoma		8 (12.2)
	Squamous cell		54 (81.8)
	Carcinoma		1 (1.5)
	High-grade dysplasia		1 (1.5)
	Not informed		2 (3)
Histologic grade			
	Grade 1		4 (6.1)
	Grade 2		22 (33.3)
	Grade 3		2 (3)
	Undifferentiated		3 (4.5)
	Undetermined		35 (53)
Clinical staging (TNM)			
	Tumor		
		T1	4 (6.1)
		T2	23 (34.8)
		T3	28 (42.4)
		T4	5 (7.6)
		TX	6 (9.1)
	Nodes		
		N0	40 (60.6)
		N1	16 (24.2)
		N2	1 (1.5)
		NX	9 (13.6)
	Metastasis		
		M0	57 (86.4)
		MX	9 (13.6)
Neoadjuvant therapy			
	Chemotherapy		2 (3)
	Radiotherapy		2 (3)
	Radiotherapy + Chemotherapy		32 (48.5)
	None		30 (45.5)
Neoadjuvant therapy complete			
	Response		16 (44)

SD: standard deviation; SAH: systemic arterial hypertension; ASA: American Society of Anaesthesiologists; EGD: Esophagogastroduodenoscopy; DA: dental arch; EGT: esophagogastric transition.

Regarding the preoperative pathological characteristics, 50% of lesions were localized (through EGD) between 20 and 30 cm from the dental arch (DA) and 25.7% between 30 and 40 cm from DA. The main histological type was the SC (81.8%). As for the clinical TNM staging, the most frequent were T3 and T2 (42.4% and 34.8%, respectively), N0 and N1 (60.6% and 24.2%, respectively), and M0 (86.3%). Of the total, 32 patients (48.4%) received the combination of NT with RXT and CT, while 30 patients (45.4%) received no NT.

### Operative outcomes

The most performed type of surgical approach was hybrid MIE with thoracoscopy in prone position and laparotomy. Only two patients (3%) underwent a totally minimally invasive approach, by videothoracoscopy and videolaparoscopy. The mean operative time was 301 minutes (±36.8) and 95.5% of cases had a complete resection and negative margins (R0). Distant metastases were not detected in this study. The average number of resected lymph nodes was 16 (±6). No patient required blood transfusion, and there were no intraoperative complications. Operative and postoperative outcomes are detailed in Table 2.

**Table 2:** Operative and postoperative outcomes.

			Sample (n=66) n (%)
Surgical approaches			
	Abdominal LP + Thoracoscopy		64 (97)
	Abdominal VLP + Thoracoscopy		2 (3)
Operative time, minutes (SD)			301 (±36.8)
R0-resection			63 (95.5)
Lymph nodes retrieved (SD)			16 (±6)
Morbidity			25 (37.9)
	Pneumonia		1 (1.5)
	Empyema Wound		3 (4.5)
	Infection		2 (3)
	Hematoma		4 (6)
	Stenosis anastomosis		2 (3)
	Vocal cord paralysis		22 (33.3)
	Fistula		18 (81.8)
		Cervical	4 (18.2)
		Mediastinal	1 (1.5)
	Chylothorax		1 (1.5)
	Gastric conduit necrosis		1 (1.5)
	Evisceration		1 (1.5)
	Stroke		1 (1.5)
	Sepsis PE		1 (1.5)
	UTI		1 (1.5)
Mortality			8 (12.1)
Reoperation			9 (13.6)
Length of stay, days (SD)			19.93 (±15.69)

SD: standard deviation; LP: laparotomy; VLP: videolaparoscopy; PE: pulmonary embolism; UTI: urinary tract infection; R0: resection.

### Postoperative outcomes

The incidence of postoperative pneumonia was 38%, and the incidence of fistula was 33.3%, with most cases of cervical anastomosis (82%), and a few of mediastinal fistula (18%). Nine patients needed to be reoperated (13.6%), four of them due to mediastinal fistula, two due to evisceration, one due to chylothorax, one due to empyema, and one due to gastric tube necrosis. The mean length of hospital stay was 19.9 days (±15.6). Four patients (6%) presented stenosis of the cervical anastomosis with treatment performed with endoscopic dilation, and two patients (3%) presented postoperative vocal cord paralysis.

Concerning the univariate analysis for fistula (Table 3), there was no statistical significance among the variables evaluated. However, in the logistic regression model of the multivariate analysis, after individually adjusting variables for the patient's age, we found that patients presented an 8.2% chance of developing fistula for each additional year of age, keeping the year of surgery constant, with a trend to statistical significance (odds ratio [OR] 1.082; 95% confidence interval [CI] 1.003–1.185; p-value [p]>0.05). Other findings on fistula are described in Table 4.

**Table 3:** Univariate analysis for fistula and pos-operative death.

	Fistula			Postoperative death		
	OR	95%CI	p-value	OR	95%CI	p-value
Neoadjuvant therapy	0.760	0.269–2.132	0.600	0.813	0.176–3.737	0.783
Neoadjuvant therapy complete response	0.245	0.032–1.244	0.115	0.356	0.017–3.129	0.393
Clinical T staging	0.848	0.485–1.423	0.541	1.440	0.694–2.936	0.311
Clinical N staging	1.076	0.728–1.556	0.699	1.733	1.101–2.759	**0.016**
Number of lymph nodes dissected	0.943	0.851–1.031	0.225	0.939	0.800–1.068	0.390
Pneumonia	5.436	1.067–40.89	0.056	6.158	1.283–44.82	**0.035**
Treatment response	1.178	0.931–1.507	0.177	1.054	0.752–1.494	0.757
Surgery year (1-year increase)	0.901	0.724–1.113	0.337	1.249	1.069–1.529	**0.014**
Patient age (1-year increase)	1.062	0.990–1.148	0.101	1.138	1.014–1.319	0.051

OR: Odds Ratio; CI: Confidence Interval.

**Table 4:** Multivariate logistic regression for fistula.

	Fistula			Adjusted for patient age (1-year increase)		
	OR	95%CI	p-value	OR	95%CI	p-value
Neoadjuvant therapy	0.769	0.266–2.210	0.623	1.062	0.990–1.148	0.103
Neoadjuvant therapy complete response	0.461	0.050–3.340	0.453	1.089	0.956–1.292	0.253
Clinical T staging	0.894	0.514–1.506	0.679	1.060	0.990–1.147	0.113
Clinical N staging	1.085	0.727–1.590	0.675	1.062	0.990–1.149	0.100
Number of lymph nodes dissected	0.933	0.841–1.021	0.153	1.070	0.998–1.160	0.073
Treatment response	1.158	0.907–1.491	0.244	1.056	0.980–1.143	0.146
Surgery year (1-year increase)	0.836	0.642–1.058	0.153	1.082	1.003–1.185	0.059

OR: odds ratio; CI: confidence interval.

In the logic regression model of the multivariate analysis for pneumonia (Table 5), the duration of surgery was associated with this outcome in the postoperative period (OR 1.026; 95%CI 1.007–1.054; p=0.022). When adjusting this variable for age, we found that, for each additional minute in the duration of surgery, the chance of the patient developing pneumonia increased by 14.8%, with a tendency to statistical significance (OR 1.148; 95%CI 1.011–1.360; p=0.062). In addition, there was statistical significance when pneumonia was associated with the year the surgery was performed (OR 0.612; 95%CI 0.372–1.920; p=0.029), increasing by 24.9% the chance of the patient developing this complication for each year of age they present at the date of the procedure (OR 1.249; 95%CI 1.069–1.529; p=0.014).

**Table 5:** Multivariate logistic regression for pneumonia.

	Pneumonia			Adjusted for patient age (1-yearincrease)		
	OR	95%CI	p-value	OR	95%CI	p-value
Tobacco	2.099	0.601–7.718	0.248	1.033	0.960–1.114	0.368
Alcohol	1.775	0.505–6.366	0.368	1.033	0.960–1.114	0.368
Neoadjuvant therapy	0.503	0.178–1.380	0.185	1.033	0.970–1.107	0.336
Neoadjuvant therapy complete response	2.278	0.346–16.31	0.392	1.066	0.941–1.234	0.342
Clinical T staging	1.002	0.486–2.101	1.000	1.033	0.970–1.108	0.337
Clinical N staging	0.914	0.512–1.553	0.746	1.033	0.970–1.108	0.337
Number of lymph nodes dissected	0.933	0.841–1.021	0.153	1.070	0.998–1.160	0.073
Surgery duration (1-minute increase)	1.026	1.007–1.054	0.022	1.148	1.011–1.360	0.062
Surgery year (1-year increase)	0.612	0.372–1.920	0.029	1.249	1.069–1.529	0.014

OR: odds ratio; CI: confidence interval

In the univariate analysis for postoperative death, pneumonia (OR 6.158, 95%CI 1.283–44.820, p=0.035) and clinical N staging (OR 1.733, CI 1.101–2.759, p=-value 0.016) presented statistically significant results; in relation to the patient's age, there was a trend towards statistical significance (OR 1.138, 95%CI 1.014–1.319, p<0.05). In contrast, in Table 6, of multivariate logistic regression for death, both clinical N staging and year of surgery presented significant statistical data (p=0.012 and p=0.029, respectively), and pneumonia presented a tendency to statistical relevance (p≈0.05). After adjusting the variables for the patient's age, we observed that tumor size (OR 1.154, 95%CI 1.022–1.352, p=0.042), the number of compromised lymph nodes (OR 1.172, 95%CI 1.028–1.400, p=0.041), the number of resected lymph nodes (OR 1.156, 95%CI 1.022–1.360, p=0.043), and the year when the surgery was performed (OR 1.249, 95%CI 1.069–1.529, p=0.014) were independent variables associated with death in these patients. This last finding corroborates the existence of a trend in the reduction of mortality of patients associated with the learning curve of our service, and since 2018, the mortality rate was 3.2% (one case) with no more deaths reported since 2019.

**Table 6:** Multivariate logistic regression for postoperative death.

	Pos-operative death			Adjusted for patient age (1-yearincrease)		
	OR	95%CI	p-value	OR	95%CI	p-value
Neoadjuvant therapy	0.907	0.186–4.532	0.901	1.137	1.013–1.319	0.053
Neoadjuvant therapy complete response	1.167	0.041–20.933	0.916	1.182	0.962–1.606	0.193
Clinical T staging	1.589	0.776–3.289	0.197	1.154	1.022–1.352	0.042
Clinical N staging	1.917	1.169–3.313	0.012	1.172	1.028–1.400	0.041
Number of lymph nodes dissected	0.919	0.775–1.048	0.259	1.156	1.022–1.360	0.043
Pneumonia	5.436	1.067–40.89	0.056	1.123	1.001–1.303	0.079
Treatment response	0.990	0.678–1.444	0.970	1.138	1.013–1.321	0.053
Surgery year (1-year increase)	0.612	0.372–0.920	0.029	1.249	1.069–1.529	0.014

OR: odds ratio; CI: confidence interval

### Mortality and survival

**Figure 1:**
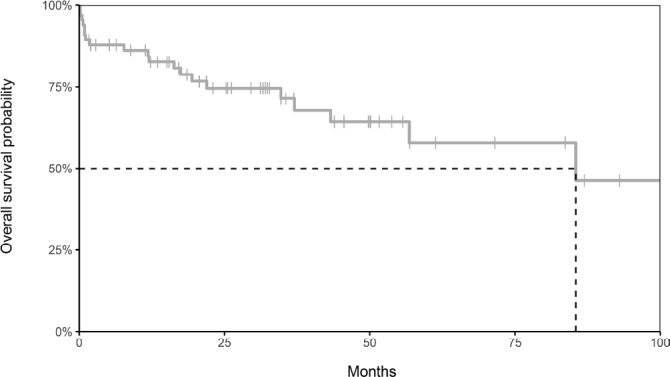
Kaplan-Meier curve of the estimated overall survival.

The incidence of death related to surgery was 12% (eight cases). In the survival analysis, the estimated 5-year overall survival (OS) was 58% in the total group (Figure 1).

**Figure 2:**
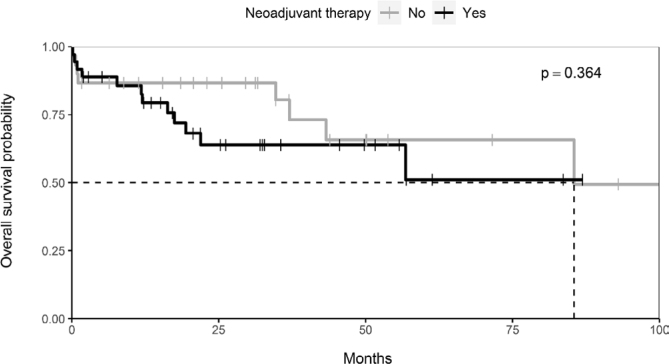
Kaplan-Meier curve for neoadjuvant therapy and overall survival.

Patients who underwent exclusive surgical treatment had an estimated 5-year OS of 66% versus 51% of those who were submitted to NT followed by surgery. *(* Figure 2 *)*

**Figure 3:**
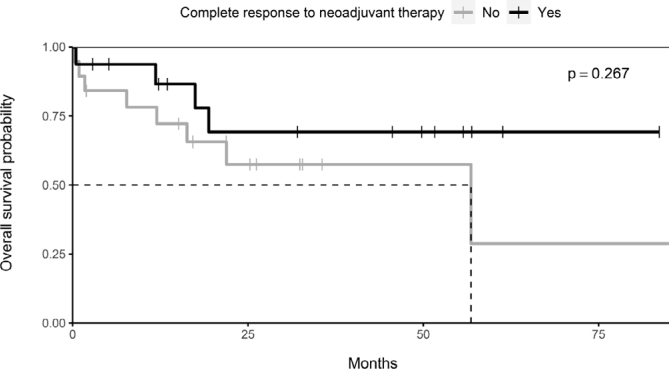
Kaplan-Meier curve for the response to neoadjuvant therapy and overall survival.

In patients who presented pathological complete response after NT, the estimated 5-year OS was 69%, against only 29% in the group with residual disease; however, this difference had no statistical significance (p=0.267). (Figure 3).

## DISCUSSION

Esophagectomy is one of the pillars in the treatment of esophageal cancer. It is a highly complex procedure, especially for an organ that crosses three anatomical compartments (neck, thorax, and abdomen) and is located very close to important mediastinal structures, with potential for early and erratic lymph node dissemination. The best approach to treat this cancer remains a subject of much discussion regarding the different types of surgical techniques described, varying according to the lesion site, the patient's clinical condition, and service experience^3–6,12,13,16–18^.

The MIE had its first results reported by Luketich et al. in 1998, and since then it has been increasingly applied, with consistent results showing reduced morbidity rates, especially pulmonary complications. Prospective studies have also demonstrated the long-term safety of the technique with overall survival and progression-free survival similar to that of open techniques. However, as in the open technique, different types of minimally invasive approaches are possible, from totally minimally invasive procedures to hybrid procedures, combining minimally invasive and conventional approaches to one compartment^3,4,12,13,16,18^.

In our service, we opted for the thoracic approach by thoracoscopy in a prone position followed by the conventional abdominal stage with cervical anastomosis using the gastric tube reconstruction as the first option, mainly because 79% of our cases were located in the thoracic esophagus and 82% were SCs. We believe that this histological type and tumors in this location require a thoracic esophagus approach under direct visualization with the possibility of adequate and safe lymph node dissection of the lesion and the entire esophagus, when intrathoracic reconstruction is not possible. It is also feasible to perform this stage of dissection with conventional materials such as electrocautery and permanent forceps, sparing the patient a thoracotomy, dismissing selective intubation, reducing lung manipulation, and enabling the performance of the procedure with only three portals^17^. For the abdominal stage, we initially chose to do it by conventional route due to limited materials available such as endostaplers and power clamps in public hospitals. Since the implementation of this approach, the postoperative mortality rate was 12%, fistula incidence was 33%, and postoperative pneumonia incidence was 38%.

In regards to postoperative death in univariate and multivariate analyses, the factors that influenced this rate were the patient's age, T and N stages, the year the procedure was performed, the extent of lymph node dissection, and postoperative development of pneumonia. Other variables such as performing NT, sex, and response to NT did not influence the increase in surgical mortality.

The association between mortality and the year when the surgery occurred showed the importance of the team's experience in performing the procedure. Each year in our service there was a 24% reduction in the chance of death with surgery, with the last death registered in March 2019, and 14 procedures were performed since then. In the last three years, the mortality of this procedure was 3.2%, with only one death recorded since 2018. These data are already well established in the literature, which shows the importance of concentrating the treatment in reference centers and with teams focused on the surgical treatment of esophageal cancer. Series has shown mortality above 10% in low-volume centers, dropping to less than 5% in centers with high volume/year^2,8,11,12,14^.

Although 80% of patients had lesions from T2 and/or N+, only 55% of patients of this study were submitted to NT with RXT and/or CT. According to the univariate and multivariate analysis, clinical lymph node status, clinical T staging, and extent of lymph node dissection were associated with a higher risk of death postoperatively, suggesting that the greater the extent of the disease and of the surgery, the greater the chance of death in the postoperative period. The complete response rate on the surgical specimen after RXT and CT was 44%, similar to the results described in the Cross Trial, 49% for SC^1,21^. In these patients, as observed in the literature, the 5-year OS rate was 69%. Analyzing specifically this subgroup of patients, the surgical mortality rate was 6.2%, lower than that of the total group that was 12%; however, these data were not significant, in the univariate analysis. We believe that the better results observed in these patients were related to the good response of the tumor to NT, which probably allowed the patient to present better clinical conditions when submitted to the surgical procedure.

Another data analyzed was the interval between the end of the NT and the surgical procedure. Several studies suggested that the greater this interval, the greater the morbidity and mortality of surgery due to the deleterious effects of RXT and the greater the technical difficulty^10,15,22,23^. In our study, the mean interval was 174 days, ranging from 45 to 720 days, much longer than the recommended 28 to 84 days. Nonetheless, this interval was not associated with increased surgical morbidity or mortality.

The incidence of pneumonia in the postoperative period was directly related to the duration of the procedure and the year in which the surgery was performed. It was also significant in the univariate analysis of postoperative death. Pneumonia is one of the main complications described after esophagectomy, and several studies have shown the importance of minimally invasive surgery to reduce this incidence. Our data indicate the importance of the experience acquired over the years with MIE in reducing the incidence of postoperative pneumonia.

Assessing the incidence of fistula in the postoperative period, we observed a downward trend of this outcome over the years, corroborating the improvement in surgical results with the increase in the service experience. In all cases, an anastomosis was performed in the cervical region, which is associated to postoperative fistula due to tension of the esophagogastric anastomosis and ischemia of the proximal part of the gastric tube. However, the management of this type of fistula is simpler than a fistula from mediastinal anastomosis, often being treated conservatively with local drainage, antibiotics, and appropriated diet until resolution, without the need for reapproach or use of endoscopic stents^20^. In our study, reoperation was indicated in nine cases (13%), a rate lower than that of the fistula (22 patients, 33%), corroborating the possibility of conservative treatment in most patients. Another significant finding herein was that NT with RXT and/or CT as well as a long interval between the end of NT and surgery were not associated with an increased fistula rate. Several studies have shown that NT does not seem to increase the rate of fistula^10,15,21–23^.

The presence of a fistula was also an important factor in postoperative surgical mortality: of the eight patients who died, seven had fistula; and of these seven, four were guided to mediastinum, showing the greater severity of this type of fistula. Despite the high rate of fistula, only four patients (6%) presented anastomotic stenosis, which was all solved with endoscopic dilation, and only two patients (3%) presented prolonged vocal cord paralysis.

Finally, the profile of patients treated in our service is people of low socioeconomic status with difficulty accessing adequate nutritional therapies in preoperative period. We believe that this may also have influenced the high rate of complications described in the study; however, this variable could not be analyzed due to the absence of precise information in the medical records.

## CONCLUSIONS

The present study indicated the importance of the team's experience and the concentration of the treatment of patients with esophageal cancer in reference centers, allowing to significantly improve the postoperative outcomes of pneumonia, fistula, and death.
